# Book Notices

**Published:** 1868-07-15

**Authors:** 


### *BOOK NOTICES.*

LESSONS IN PHYSICAL DIAGNOSIS. By Alfred L. Loomis, M.D., Professor of the Institutes and Practice of Medicine in the Medical Department of the University of New York ; Physician to Bellevue and Charity Hospitals, etc. New York: Robert M. DeWitt, publisher, No. 13 Frankfort Street. Pp. 159.

A very excellent little book, which we take pleasure in recommending to students and the profession as containing a satisfactory *resumé* of the subject.

CIRCULAR NO. 1. WAR DEPARTMENT, SURGEON GENERAL'S OFFICE, WASHINGTON, JUNE 10, 1868. REPORT ON EPIDEMIC

### Cholera and Yellow Fever in the U. S. Army during 1867.

The most valid objections to the statistical method of observation in the field of medical science, are insufficient data and hasty generalization.

These objections do not apply to the volume whose title is quoted above. The data reported therein are furnished by more than thirty competent observers, at as many different localities, embracing an area of territory commensurate with the spread of the epidemics considered; constituting, as they do, one of the most, if not the most, valuable collections of information accessible to the general practitioner, these reports should be studied with care by every medical man interested in the solution of the problems of epidemiology.

The observations upon cholera seem to justify the following conclusions:

1st. That cholera in its movement follows lines of travel, and especially those of infected troops, and hence that it is transported, by preference, by human agency.

2nd. That troops under rigid hygienic regulations, designed to protect them against another danger (yellow fever) enjoyed an immunity against cholera not participated in by citizens surrounding them, not so protected.

These conclusions diminish the agency of *atmospheric transportation* to a minimum.

3rd. Its reappearance, without fresh importation, after a disappearance for six months, indicates a dormant vitality, or retained viability of the cholera element, under unfavorable atmospheric conditions, needing only a modification of these conditions to re-develop it into activity.

4th. The ratio of sick to population differed widely in different localities, whilst the ratio of mortality to the sick was nearly uniform every where, indicating the necessity of other elements than the specific poison *per se*, viz.: favorable personal and atmospheric conditions, to combine for the production of the result, infection, either of which elements being absent, the specific poison is inert. Were direct testimony wanting, the above would supply a strong inferential argument in favor of a strict system of hygienic and sanitary regulations.

5th. It is transportable by means of corpses.

6th. It is retained and preserved in a state of potential activity in localities once infected, and subsequently abandoned.

7th. A definite period of incubation of ten to fourteen days is observed.

These deductions will be (perhaps) useful practically here as elsewhere, before, during, or after cholera epidemics. *Verbum sap.* (Board of Health) *sat.*

From a consideration of the reports on yellow fever, the following conclusions appear to be justified:

1. That the epidemic is exotic.

2. That the proximity of tide water is one of the essentials to its origin and self-developed activity.

Note.—Incidents occurring during other epidemics seem to modify this conclusion.

3. That of two foreign sources of importation, one, viz.: Mexico, appears to have supplied the poison in a more virulent form than the other — Cuba, (this conclusion probably applies peculiarly to this epidemic) the difference in the ratio of mortality being 12.5 per cent. (viz.: 40 and 28.5 respectively).

4. That the immunity conferred by acclimatization may be lost, to a great extent, by subsequent subjection to different climatic influences.

5. That the African enjoys a remarkable immunity against this, in common with all other malarial diseases, and, moreover, that his immunity against death is even greater than that against infection. The proportion of cases amongst the whites being 80, and of deaths 25 per cent., whilst amongst the colored these proportions were 50 and 7 per cent., respectively.

6. That the beneficial effects of a rigid quarantine as a preventive of yellow fever infection, have been demonstrated most satisfactorily.

7. That there is a period of incubation, which may be extended to twenty days.

It is to be regretted that some definite conclusions can not be derived from these reports, upon the subject of the treatment of these two diseases. For these we must be content to await the accumulation of still further evidence, before attempting to generalize.

In the mean time, it is to be hoped that the Surgeon General will continue to give to the professional world the results of the observations in his Department, in a form similar to the work now under consideration. W. H.

---

Important Information.—A distinguished medical teacher in the late Medical Department of the University of Michigan denounced in strong terms before the class the gross negligence, and, indeed, criminality, which would endanger life in *post partum hæmorrhage*. Nothing is easier, he asserts, than to introduce the hand into the vagina and compress the aorta on the promontory of the sacrum, which will infallibly control the flow. It is fair to add that some of the younger members of the class doubted — a peculiar action of mind suggested by the idiosyncrasy of their accomplished Professor of Obstetrics. The first mentioned Professor, it is said, formerly undertook the cure of mammary cancer by manipulation of the sound breast. *Counter-irritation* by SEARING.

